# Endocrine disrupting chemicals: effects on pituitary, thyroid and adrenal glands

**DOI:** 10.1007/s12020-022-03076-x

**Published:** 2022-05-23

**Authors:** Filippo Egalini, Lorenzo Marinelli, Mattia Rossi, Giovanna Motta, Nunzia Prencipe, Ruth Rossetto Giaccherino, Loredana Pagano, Silvia Grottoli, Roberta Giordano

**Affiliations:** 1 Endocrinology, Diabetes and Metabolism, Department of Medical Sciences, University of Turin, Corso Dogliotti 14, 10126 Turin, Italy; 2 Department of Biological and Clinical Science, University of Turin, Regione Gonzole 10, 10043 Orbassano (TO), Italy

**Keywords:** Endocrine disrupting chemicals, Endocrine disruptors, Pituitary gland, Thyroid gland, Adrenal gland

## Abstract

**Background:**

In recent years, scientific research has increasingly focused on Endocrine Disrupting Chemicals (EDCs) and demonstrated their relevant role in the functional impairment of endocrine glands. This induced regulatory authorities to ban some of these compounds and to carefully investigate others in order to prevent EDCs-related conditions. As a result, we witnessed a growing awareness and interest on this topic.

**Aims:**

This paper aims to summarize current evidence regarding the detrimental effects of EDCs on pivotal endocrine glands like pituitary, thyroid and adrenal ones. Particularly, we directed our attention on the known and the hypothesized mechanisms of endocrine dysfunction brought by EDCs. We also gave a glimpse on recent findings from pioneering studies that could in the future shed a light on the pathophysiology of well-known, but poorly understood, endocrine diseases like hormone-producing adenomas.

**Conclusions:**

Although intriguing, studies on endocrine dysfunctions brought by EDCs are challenging, in particular when investigating long-term effects of EDCs on humans. However, undoubtedly, it represents a new intriguing field of science research.

## Background

Endocrine disrupting chemicals (EDCs) are defined as “exogenous chemicals or mixture of chemicals that interfere with any aspect of hormone action” [1]. Their extensive application in several fields (agricultural, industrial, residential and pharmaceutical), their ability to contaminate human body through virtually every route (inhalation, digestion and transdermal) [2, 3] and to accumulate even for years in the adipose tissue [4, 5], easily explains the danger that the chronic exposure, to even small doses, poses.

Given the complexity of the whole endocrine system and the variety of the involved substances, infinite are the possible levels interfered by EDCs: synthesis, secretion, transport, metabolism, or elimination of endogenous hormones [2, 6–11]. More recently, literature has focused on their supposed direct action on hormonal receptors and/or genomic expression [12]. Indeed, several mechanisms of action appear to be involved in endocrine disruption. They can act as total, partial, or inverted agonists or as antagonists for endocrine nuclear receptors [6, 10, 11], and even perform epigenetic changes, such as DNA methylation and/or acetylation and histone modifications [13, 14].

For comparable reasons, endocrine impairment induced by EDCs can express in a wide range of consequences: hormonal secretion, cell proliferation and cancer, growth, metabolism, sexual development, circadian clocks, and even, cognitive functions, neurodevelopment and behavior, mostly following recent literature on pre/postnatal exposure [1].

Indeed, several studies carried out during pregnancy and lactation, show that EDCs exert influence in both the exposed individual and in their offspring. Not only, EDCs damage during development, and not necessarily after conception, seems also able to transfer to future generations, through a process known as transgenerational inheritance [15].

All this can be explicated by the deleterious effects of EDCs on three of the main human endocrine axes: hypothalamus-pituitary, adrenal, and thyroid glands.

Table 1 presents some information about the main various substances you will find throughout this dissertation [16].

**Table 1:** Main EDCs and their applications [16]

Applications of EDCs	
	Applications
Environmental pollutants	
Dioxin	Non-man made, unintentional byproducts of industrial production and combustion
Polycyclic aromatic hydrocarbons	
Flame retardants	
Polychlorinated biphenyls (PCBs)	Added to textile, electronic, and construction materials and foam products to make them difficult to burn
Polybrominated diphenyl ethers (PBDEs)	
Repellents	
Perfluoroalkyls (PFAS: PFOA and PFOS)	Used to protect products like carpet and fabric, as a coating for paper and cardboard packaging, and in some firefighting foams
Plastics	
Bisphenol A	Found in a wide variety of domestic applications, including baby bottles and food packaging
Phthalates	
Methacrylonitrile	
Pesticides and fungicides	
Dichlorodiphenyltrichloroethane (DDT)	Insecticide, mainly used against Anopheles mosquito in the eradication of malaria
Chlordane	Insect control of stored food and clothing; as well as termite control, carpenter ants and wood beetle
Endosulphan	Pesticide used widely on crops like cashew, cotton, tea, paddy, fruits and others
Tributyltin chloride	Fungicide agents in textiles and paper, wood pulp and paper mill systems, breweries
Aerosol	
Carbon tetrachloride	Refrigerant fluid, propellants for aerosol cans, pesticides, cleaning fluid and degreasing agent, in fire extinguishers, and in spot removers. These uses are banRed, and it is now only used in some industrial applications

## Pituitary gland

The pituitary gland is a potential target of EDCs, which can result in an alteration of the pituitary hormone-releasing patterns. The hypophysis seems to be vulnerable through direct and hypothalamic-mediated processes exerted by these compounds [1, 17]. However, EDCs mechanisms of action are still not fully figured out: over the entire lifespan of the individual, they could interfere with endogenous hormonal function, affecting the homeostatic system, or alter the genomic expression, e.g., through DNA methylation [1, 12].

A growing body of evidence is suggesting that EDCs can have an influence on tumorigenesis. Researchers described a link between EDCs and cancer burden, particularly with testicular, breast and prostate cancer [1]. Pituitary gland seems to be a potential target of these compounds, too. First epidemiological studies reported a higher incidence of pituitary adenomas due to previous exposure to dioxin [18] and a higher incidence of growth hormone- (GH-)secreting adenomas in a highly industrialized area nearby Messina, Italy [19]. Moreover, in vitro studies succeeded in demonstrating correlations between pollutants and stimulation of pituitary cells: benzene and phthalates increased cell proliferation via a deregulation of aryl hydrocarbon receptor (AHR) and AHR-interacting protein (AIP) [20], a tumor suppressor pathway that seems to be involved with other xenobiotics, such as polycyclic aromatic hydrocarbons and PCBs [21]. The involvement of AIP seems to play a key role: in fact, previous studies had already linked AIP gene mutations with familial isolated pituitary adenoma syndrome, familial somatotropinomas, and with apparently sporadic acromegaly [22, 23]. Moreover, in clinical practice AIP gene mutations are associated with an aggressive disease phenotype, which is less responsive to conventional medical treatment, as somatostatin analogues [19].

In addition, another influence of EDCs on the hypothalamic–pituitary–somatotropic axis can be hypothesized: an increase in GH secretion was reported for octyl- and nonyl-phenols, BPA [24], benzene, phthalates, and PCBs [25]. Furthermore, estrogens exert a direct stimulation of GH and prolactin (PRL) release [26, 27]; consequently, a potential increase in GH levels could be hypothesized for those EDCs with estrogenic activity, such as diethylstilbestrol and DDT [1].

Lactotroph cells also resulted to be susceptible to EDCs: the known estrogenic activity of BPA, and a similar activity performed by two pesticides (endosulphan and chlordane) seemed to induce PRL secretion both in vitro and in vivo [28–30]. Even metal ions with analogous endocrinological influence, the so-called “metalloestrogens” [31], such as lead and chromium, resulted to be positively associated with PRL release in humans [32–34]. However, this evidence resulted not to be conclusive: further studies documented that cadmium, mercury, molybdenum, and lead were inversely associated with PRL levels [34–37].

The impact made by EDCs on the hypothalamic-pituitary-thyroid axis is still unclear. In animals, reduced serum TSH and not univocal fT3 and fT4 responses were reported after exposition to pesticides and fungicides [38–40]; tributyltin chloride, however, was associated with increased TSH and diminished fT3 and fT4 serum levels [41, 42]. Even in humans, there were interesting findings: workers exposed to cadmium showed metal urinary concentrations to be directly correlated with serum TSH and inversely with fT3 and fT4 [43]. Furthermore, some studies demonstrated a lasting effect from mother to child: they reported a positive association between maternal and children blood levels of TSH and PFAS, PCBs, or dioxin [44–46], while BPA urinary levels in pregnant women resulted to be inversely associated with serum TSH, but not with fT3 and fT4 levels of newborns [47, 48].

Direct hypothalamic-pituitary-adrenal axis involvement with EDCs is still a matter of debate due to the lack of evidence, especially in human species. A few animal studies reported a direct suppression of this axis through a reduction in adrenocorticotrophic hormone (ACTH) and corticosterone levels after PCBs exposition [49], chiefly in female rats [50], or indirectly through reduction of hypothalamic corticosterone-releasing-factor mRNA [51]. BPA increased ACTH and corticosterone levels in male, but not in female rats [52].

Rising evidence outlines how EDCs can have implications for the neurohypophysis, which acts like a storage site of hypothalamic released hormone vasopressin (AVP) and oxytocin. In particular, in mammalians BPA peri-natal exposure was associated with upregulation of oxytocin release, both in males [53] and females [54] while no different number of oxytocin neurons was found after prenatal exposition to PCBs [55]. AVP function was reported to be influenced by BPA [54, 56] and PBDE exposure increased the number of AVP releasing neurons [57].

Wide is the literature about the influence of EDCs on hypothalamic-pituitary-gonadal axis. An intriguing subtopic is represented by puberty disruption. Puberty is a critical time orchestrated by a pulsatile release of hypothalamic gonadotropin-releasing hormone (GnRH) leading to an episodic systemic secretion of luteinizing hormone (LH) and follicle stimulating hormone (FSH) from the anterior pituitary gland [58]. This system is mainly regulated by hypothalamic kisspeptin neurons, which are usually inhibited during childhood [59]. These neurons and pituitary cells are sensitive to endocrine influences during fetal, neonatal, and juvenile development [60]. In literature, there are reports of early menarche in daughters of women exposed to pesticides and phytoestrogens [61, 62] and in girls consuming soy or exposed to PBDE [62–64] during infancy [65]. Finally, several animal models of EDCs exposure have confirmed their role on pubertal timing, even if the mechanisms of action are still not completely elucidated [66]. In mammalian, BPA seems to exert a stimulatory effect on kisspeptin-GnRH system [67–69] while other claim that BPA [70–72], PCBs [73], sewage sludge [74] can have an inhibitory effect. Moreover, mixed effects (BPA [75], PCBs [76], phthalates [77]) or no effect on kisspeptin-GnRH system are also reported [73].

Finally, it is intriguing to consider the perturbation exerted by EDCs on circadian rhythms, which synchronize cell functions with the external light-dark cycle. Circadian clocks are highly conserved, endogenous time-keeping mechanisms that generate self-sustained oscillations with an approximately 24-h period [78]. The main characters of this regulation system are the clock genes; in the anterior hypothalamus, they encode for transcription factor like BMAL-CLOCK1 [79] that drives rhythmic expression of many clock-controlled genes, even in humans [80]. Alterations of this daily pattern seem to have potential detrimental effects on human health, including infertility, cancer [80–82], and also disruption of pituitary hormone release patterns [81, 83–86]. In fact, exposure to cadmium can disrupt rhythmic 24-h release of PRL [87], BPA and dioxin can alter the expression of BMAL gene [88, 89] and even tributyltin seems to have a harmful effect on this system [90]. Moreover, gestational exposure to PCBs disrupted the normal BMAL expression pattern [91–93].

The systematic study of the whole series of alterations to the pituitary gland made by EDCs results to be challenging, due to the high complexity of this gland. As we have seen, the various hypothalamic-pituitary axes can be involved in varying degrees. In conclusion, it is still a matter of debate the comprehension of the entity of EDCs burden on the etiopathogenesis of pituitary diseases.

## Thyroid gland

An extraordinary variety of substances can interfere with the normal functioning of the thyroid gland. Given the complexity of the hypothalamus-pituitary-thyroid axis, the great amount of involved mechanisms is not surprising.

Historically involved processes include the inhibition of the sodium/iodine symporter (NIS) and of the thyroid-peroxidase enzyme (TPO), widely described for some ions that pollute water basins (nitrates, perchlorates) and cigarette smoke (thiocyanate) [94]. These are, among other substances, known as goitrogens in light of their ability to increase the volume of the gland, through the proliferative stimulus of lower thyroid hormones (THs).

Recent studies showed how often pre/postnatal exposure to EDCs is consistently associated to children neurodevelopment impairment, and although conclusive evidence about specific differences for age and genders still lacks, some studies seem to point out a higher susceptibility in young boys and an effect lasting up to 7 years of age or even until adulthood [95, 96]. It is hard to pinpoint the precise mechanisms between endocrine disruption and neurodevelopmental outcome because most of these epidemiological studies lacked in complete endocrine profiles. It is well-known that THs are central in normal cerebral development. Even transient and mild hypothyroidism in pregnancy is also associated with cognitive/neurobehavioral in the offspring (e.g., 3.9 IQ score loss in association with maternal fT4 < 2.5 mUI/L) [97]. Consequently, it is absolutely established the causal burden of goitrogens. Yet, such an impairment does not always coincide with a strong negative correlation with serum levels of THs [95, 98–101]. This observation shifted the focus of research on a downstream action. Particularly, it is now recognized that some chemicals, mainly flame retardants (PCBs, PBDEs), plasticizers (phthalates, BPA) and certain pesticides, can directly interfere with thyroid hormone receptors (THRs) and their transcriptional activity at several levels, which also made them suspect of increasing the risk of thyroid cancer [102].

Studies suggested an association of prenatal and infantile exposure to PCBs with inferior IQs in children [103], but less consistent is the evidence about correlation with T4 and TSH levels [104]. Interestingly, PCBs seem to be among the few substances capable of directly interfere with THRs, which added to the transport-globulin mechanism, could explain the direct cortical antigrowth action [105, 106]. The same, with minor difference, can be said of PBDEs, the employment of which raised with the abandonment of PCBs, in the 80 s [102, 107–109].

Research has shown that phthalates carry out their anti-thyroid action via a by-product of a gram-negative bacteria processing, which behave as a TPO inhibitor. They were also classified as “possibly carcinogenic to human” (Class 2B) by IARC [102, 110].

Recent studies found that BPA has a negative correlation with T4 levels in several large population-based studies [111–114]. Its action can be associated to its binding with thyroxin binding globulin (TBG) and Transthyretin (TTR) [115], to the interference with THRs [116] and, moreover, to an increased the expression of Dio1 gene and Ugt1ab, and consequently to an augmented catabolism of TH [117, 118]. BPA is known to stimulate the proto-oncogenic estrogen receptor (mER) and to activate nuclear factor kB (NF-kB), a transcription factor involved in development of thyroid cancer. Yet, studies investigating the association with thyroid cancer are limited [119, 120].

Full comprehension of this process is still far from reach and there are multiple factors being suggested: expression of THRs (Phthalates and BPA [121, 122]), interaction of the complex Retinoid X receptor (RXR) and THR with T3-response elements (TREs) (PCB [123]), recruitment/release of corepressors (BPA [9]), T3 binding to receptor (PBDEs, phthalates [124, 125]) and recruitment of coactivators (PCBs [126]).

The extent of how far environment and chemicals can interfere with thyroid gland functioning will always be a puzzle, because of the infinite possible mechanisms and the challenge of reaching superior quality evidence. Thyroid should be an example, though, of how such topics can have a great clinical and practical rebound. It is, consequently, of primary importance to obtain full disclosure on this subject and to design increasingly rigorous studies. Researchers’ efforts should today focus on new and barely explored fields of study, like other mechanisms than the decrease in THs blood levels and especially on the direct effect on molecular pathways and the interference with DNA transcription.

## Adrenal gland

The human adrenal gland is a complex endocrine organ that produces both steroid hormones (glucocorticoids, mineralocorticoids and androgens) and amino acid-derived hormones (epinephrine and norepinephrine). Adrenal functional impairment can lead to both insufficiency and overproduction of hormones eliciting Addison’s disease (primary hypo-adrenalism), Cushing’s syndrome (hypercortisolism), Conn’s disease (primary hyperaldosteronism) as well as adrenal androgen excess, which can have a role in premature adrenarche and adrenogenital syndromes in children and in hirsutism and infertility in women. For what refers to adrenal medulla, a catecholamine-secreting lesion (pheochromocytoma) may give rise to severe signs and symptoms because of the augmented sympathetic activity.

Sometimes hormones overproduction can underlie hormone-producing adenomas or carcinomas and might result from exposure to drugs or exogenous EDCs [127].

In fact, recearchers demonstrated that the adrenal gland is the most frequently observed site of endocrine lesions as it is particularly sensitive to toxic assault [128]. There is a wide number of chemicals with recorded in vitro and in vivo adrenal effects covering most of their classes: pesticides, plasticizers, dioxins, PCBs and polycyclic aromatic hydrocarbons [128–133].

For what refers to pathophysiology, these EDCs influence steroid biosynthesis and metabolism either as inhibitors or rarely as activators of key enzymes, or on the level of the respective enzyme expression.

As previously stated, the adrenal gland is particularly vulnerable to toxicants and there are several causes. First, there are multiple targets for toxicological assault such as receptors, transcription factors and enzymes [134]. For instance, hydroxysteroids dehydrogenases are targets for BPA that may affect their activity as well as their expression [135]. Another target are sulfotransferases that are inhibited by phthalates, chlorinated phenols and also by some phytoestrogens [136, 137].

Second, during steroid hydroxylation reactions, reactive oxygen species (ROS) are generated causing oxidative stress. It has been shown that imbalance in redox balance is implicated in impaired adrenal steroidogenesis and, more specifically, in several potentially lethal adrenal disorders including X-linked adrenoleukodystrophy, triple A syndrome and most recently familial glucocorticoid deficiency [138]. This imbalance can be brought up again by BPA causing the inhibition of the anti-oxidant enzymes superoxide dismutase, catalase, glutathione reductase, and glutathione peroxidase [139].

Third, the adrenal cortex has specific mechanisms for the selective uptake of lipoproteins, which are subsequently stored in the large pools of esterified lipid. Studies revealed that adrenal cells can take up and concentrate a wide number of toxic agents, including DDT metabolites, methacrylonitrile, and PCB metabolites. Particularly, it seems that these chemicals could remain inactive within the adrenal tissue until a period of particularly high demand for adrenal steroids, when they may be mobilized and cause damage [140–142].

Another important aspect is the potential of the adrenal gland for lipid peroxidation, due to the high content of unsaturated fatty acids in its membrane. This process is implicated in the toxic effects of carbon tetrachloride on this tissue [143].

In addition, researchers reported that unlike other vertebrates such as fish and birds, the human adrenal gland is highly vascularized, and this rich blood supply facilitates the delivery of toxins and metabolic substrates as well as the efficient removal of steroid products [144].

Moreover, this endocrine organ has a high content of enzymes of the CYP family that have the potential for bioactivation of toxins that can cause a relevant damage to the gland [145]. In particular, CYPs are well known as biocatalysts for the transformation of diverse pollutants, including pesticides and polycyclic aromatic hydrocarbons [146–148].

For the sake of completeness, it is necessary to highlight that current evidence of the influence of EDCs in the adrenal gland is mainly driven by studies focusing on the adrenal cortex. In fact, there are only a few studies on adrenal medulla reporting its impaired development in rats after pre-natal and post-natal exposure to DDT [149, 150].

Although the number of studies focusing on the effects of EDCs on animals is quite consistent, the evidence in humans is lacking. One reason for that is the low availability of human adrenal cells for researchers [145].

This is a crucial issue as studies demonstrated that there are several differences between the adrenal grands of animals and humans (anatomical, physiological, etc…). Moreover, even a partial impairment of proper adrenal function may have severe consequences on human health, a very challenging aspect to investigate in animals.

These subclinical and latent dysfunctions can be the result of the bioaccumulation of chemicals that might generate clinical effects only after several years of constant low-dose exposure [151]. In this light, recently researchers hypothesized that hormone-secreting adrenal adenomas could have a greater content of EDCs. A pioneering study by Fommei et al, despite being small-sized, reported a significantly higher concentration for α-, β-, and γ- Hexachlorocyclohexane (HCH) Hexachlorobenzene (HCB) and for PCBs in aldosterone-producing adenomas than in the normal cortex [152]. This aspect should be the focus of long-term ad hoc studies. Moreover, it is desirable a wider availability of human adrenal cells.

## Conclusions

The striking results of scientific studies on EDCs and their detrimental effects on the endocrine system opened a new and intriguing field of research. In particular, investigators reported EDCs-related dysfunction of pituitary, thyroid and adrenal glands with supporting evidence. In recent years, researchers focused on the effects of bio-accumulation of chemicals and the possible role of this process on the formation of non-functioning or hormone-producing adenomas. However, we still require support for this hypothesis, so we encourage to conduct long-term ad hoc studies with a higher sample size. In addition, it is necessary to make human endocrine cells available for pre-clinical studies. Undoubtedly, we are just at the beginning of a long journey that will definitely give us new insights over the years. This research could prevent EDCs-related endocrine dysfunctions, providing robust evidence to the regulatory authorities to allow them to promote the use of safer compounds and to phase-out hazardous chemicals.
